# Efficacy and safety of CalliSpheres drug-eluting bead transarterial chemoembolization for unresectable gastrointestinal stromal tumor liver metastases after failure of targeted therapy: a multicenter retrospective study

**DOI:** 10.3389/fonc.2026.1784905

**Published:** 2026-07-17

**Authors:** Song Liu, Ting Chen, Shunhang Yin, Guangji Yu, Qingdong Wang, Long Li, Guangsheng Zhao, Lei Zhang, Buqiang Zhuang

**Affiliations:** 1 Cancer Interventional Center, Linyi Cancer Hospital, Linyi, Shandong, China; 2 Dalian Medical University, Dalian, Liaoning, China; 3 General Surgery Department, Linyi Cancer Hospital, Linyi, Shandong, China; 4 Cancer Interventional Center/Minimally invasive Tumor Interventional Center, Affiliated Zhongshan Hospital of Dalian University, Dalian, Liaoning, China; 5 Anorectal Surgery, Affiliated Zhongshan Hospital of Dalian University, Dalian, Liaoning, China; 6 Department of Interventional Radiology, The Affiliated Hospital of Xuzhou Medical University, Xuzhou, Jiangsu, China

**Keywords:** drug-eluting beads, gastrointestinal stromal tumors, liver metastases, targeted therapy, transarterial chemoembolization

## Abstract

**Objective:**

To evaluate the clinical efficacy and safety of CalliSpheres drug-eluting bead transarterial chemoembolization (DEB-TACE) in patients with unresectable gastrointestinal stromal tumor (GIST) liver metastases after the failure of targeted therapy.

**Methods:**

77 patients with unresectable GIST liver metastases who had failed at least two lines of targeted therapy were retrospectively enrolled from three centers between January 2022 to December 2024. Based on their treatment regimen, patients were divided into an observation group (n=34) and a control group (n=43). The control group continued receiving tyrosine kinase inhibitors (TKIs) or best supportive care (BSC). The observation group underwent DEB-TACE followed by TKI therapy or BSC. Tumor response was assessed using the modified Response Evaluation Criteria in Solid Tumors (mRECIST). Overall survival (OS), progression-free survival (PFS), and adverse events during treatment were recorded and analyzed.

**Results:**

At the first efficacy evaluation, the observation group exhibited an objective response rate (ORR) of 94.12% (10 complete response [CR], 22 partial response [PR]) and a disease control rate (DCR) of 100%. In the control group, the ORR was 4.65% (2 PR) and the DCR was 39.53%. The median PFS (liver) was significantly longer in the observation group (11.0 months) than in the control group (5.0 months; P < 0.001). The median OS was also longer in the observation group (18.0 months vs. 12.0 months; P < 0.001). Prognostic analysis indicated that better baseline liver function, fewer prior lines of therapy, and DEB-TACE treatment were associated with improved outcomes. Adverse events in the observation group were primarily related to post-embolization syndrome and transient liver injury, with no increase in targeted therapy-related toxicity.

**Conclusion:**

DEB-TACE demonstrates favorable efficacy and a manageable safety profile in patients with unresectable GIST liver metastases after targeted therapy failure, representing a safe and feasible treatment option worthy of clinical application.

## 1 Introduction

Gastrointestinal stromal tumors (GISTs) are among the most common mesenchymal tumors of the gastrointestinal tract, with an annual incidence of approximately 10–20 per million (1). The liver is the most common site of metastasis and recurrence; about 20% of patients present with liver metastases at initial diagnosis, and 55%-72% develop liver metastases upon tumor recurrence. Liver metastases are a major factor affecting patient survival (2, 3). For patients with unresectable GIST liver metastases, standard treatment involves oral tyrosine kinase inhibitors (TKIs), which have improved patient prognosis to some extent (4). However, most patients develop drug resistance during treatment, and managing unresectable GIST liver metastases after targeted therapy failure poses a significant clinical challenge. Some studies have shown that transarterial chemoembolization (TACE) has certain efficacy for GIST liver metastases after TKI treatment failure (5, 6). Commonly used embolic agents include lipiodol, polyvinyl alcohol (PVA) particles, Embosphere microspheres, and gelatin sponge particles. CalliSpheres drug-eluting beads (DEBs) are a novel domestic particulate embolic agent offering both permanent embolization and slow release of chemotherapeutic drugs. Recent studies have found that DEBs are effective in TACE for hepatocellular carcinoma (7, 8), but their application in GIST liver metastases is rare. Furthermore, no controlled studies comparing DEB-TACE with best supportive care and/or TKI reintroduction are currently available. This retrospective study analyzed data from three centers on patients with unresectable GIST liver metastases after targeted therapy failure, aiming to investigate the clinical efficacy and safety of DEB-TACE and to provide a potential treatment option for these patients.

## 2 Materials and methods

### 2.1 Patient data

Patients with unresectable GIST liver metastases who had failed more than two lines of targeted therapy, admitted to Linyi Cancer Hospital, the Affiliated Hospital of Xuzhou Medical University, and Zhongshan Hospital Affiliated to Dalian University between January 2022 to December 2024, were enrolled. All patients who met the criteria were consecutively included. Inclusion criteria: (1) Liver metastasis confirmed by liver biopsy pathology and receipt of at least one cycle of DEB-TACE; (2) Age ≥18 years; (3) Performance status (PS) score of 0-2; (4) Child-Pugh class A or B, without significant cardiac or pulmonary dysfunction. Exclusion criteria: (1) Severe dysfunction of major organs; (2) PS score >2; (3) Uncontrolled extrahepatic metastatic lesions. Ultimately, 77 patients were included and divided into an observation group (n=34) and a control group (n=43) based on treatment regimen. No statistically significant differences were observed between the two groups in terms of age, gender, tumor number, tumor origin, liver function grade, or ECOG score (P > 0.05). Details are shown in **Table 1**.

**Table 1:** Comparison of baseline characteristics between the two groups.

Clinical characteristic	Observation group (n=34)	Control group (n=43)	P value
Age (Mean years)	53.57±10.63	55.11±11.07	0.269
Gender			0.885
Male	24	31	
Female	10	12	
Tumor location			0.928
Stomach	13	16	
Small intestine	20	25	
Colorectum	1	2	
Tumor number			0.926
≤3	13	16	
>3	21	27	
Tumor Size (cm)			0.938
≤5	10	13	
>5	24	30	
Extrahepatic Metastasis			0.971
Yes	7	9	
No	27	34	
Child-Pugh Grade			0.985
A	30	38	
B	4	5	
ECOG Score			0.926
0	21	27	
1~2	13	16	
Prior Lines of Therapy			0.987
Second-line	5	6	
Third-line	16	21	
Fourth-line	13	16	
Reintroduced TKI			0.922
Regorafenib	5	6	
Ripretinib	25	32	

ECOG, Eastern Cooperative Oncology Group; TKI, tyrosine kinase inhibitor.

### 2.2 Treatment methods

#### 2.2.1 Observation group

(1) DEB-TACE Procedure: The right femoral artery was punctured using the Seldinger technique. A Terumo peripheral angiography (RH) catheter was introduced. Conventional celiac trunk and common hepatic artery angiography were performed. Based on the location, size, and completeness of tumor staining, angiography of potential tumor-feeding arteries (e.g., phrenic artery, superior mesenteric artery, left gastric artery, right renal artery) was conducted sequentially to identify all tumor-feeding arteries. After super-selecting the microcatheter into the target artery, pre-configured CalliSpheres drug-eluting beads (microsphere diameter: 100-300 μm/300-500 μm; loaded drug: Epirubicin 60–80 mg) were slowly injected until contrast agent flow stagnated. After waiting for 5 minutes, angiography was repeated. Embolization was continued until tumor staining completely disappeared; if residual staining was present, additional embolization was performed. (2) Postoperative Management: Conventional postoperative treatments included liver protection, antiemetics, analgesia, and fluid replacement. Thirty patients received reintroduced targeted therapy during TACE intervals: 5 patients with second-line progression received Regorafenib; 16 patients with third-line progression received Ripretinib; 9 patients with fourth-line progression continued Ripretinib; the remaining 4 patients received BSC.

#### 2.2.2 Control group

Six patients with second-line progression received Regorafenib; 21 patients with third-line progression received Ripretinib; 11 patients with fourth-line progression continued Ripretinib; the remaining 5 patients received BSC.

### 2.3 Efficacy evaluation and adverse event monitoring

All patients underwent contrast-enhanced upper abdominal CT/MRI every 2–3 months after treatment to observe changes in intrahepatic tumor size, degree of necrosis, and the appearance of new lesions. Tumor response was evaluated using the modified Response Evaluation Criteria in Solid Tumors (mRECIST). According to mRECIST, complete response (CR) was defined as the disappearance of any intratumoral arterial enhancement in all target lesions; partial response (PR) was defined as at least a 30% decrease in the sum of diameters of viable (contrast enhancement in the arterial phase) target lesions, taking as reference the baseline sum of the diameters of target lesions; progressive disease (PD) was defined as an increase of at least 20% in the sum of the diameters of viable (enhancing) target lesions, taking as reference the smallest sum of the diameters of viable (enhancing) target lesions recorded since the treatment started; stable disease (SD) was defined as any cases that did not qualify for either PR or PD. For the observation group, tumor necrosis was closely monitored to comprehensively assess the interventional effect and determine the need for repeat intervention. Patients achieving CR entered clinical follow-up. Those with PR, SD, or PD within one treatment session received repeat treatment, provided liver function returned to baseline levels before repeat TACE. Patients who still had PD after two DEB-TACE sessions did not receive further TACE with this technique. Objective response rate (ORR) = (CR + PR)/total cases × 100%. Disease control rate (DCR) = (CR + PR + SD)/total cases × 100%. Progression-free survival (PFS) and overall survival (OS) were recorded. OS was defined as the time from the first interventional treatment to death or the last follow-up. PFS was defined as the time from the first interventional treatment to disease progression, death, or the last follow-up. Adverse events were graded according to the National Cancer Institute Common Terminology Criteria for Adverse Events (CTCAE) version 5.0 (Grades 0-4). Follow-up was conducted via telephone, WeChat inquiry, outpatient, or inpatient review until October 31, 2025.

### 2.4 Statistical analysis

Statistical analysis was performed using SPSS software (version 20.0). Categorical data were presented as numbers and percentages, and compared between groups using Fisher’s exact test. Quantitative data conforming to a normal distribution were expressed as mean ± standard deviation x ± s and compared using independent samples t-test. For baseline characteristics, Fisher’s exact test was used for all categorical variables, and the independent samples t-test was used for continuous variables. Survival analysis was performed using the Kaplan-Meier method, and survival rates were compared using the log-rank test. Variables with a P-value < 0.05 in the univariate analysis were entered into the multivariate Cox proportional hazards regression model to identify independent prognostic factors. A P value < 0.05 was considered statistically significant.

## 3 Results

### 3.1 Short-term efficacy

Digital subtraction angiography (DSA) during the intervention in the observation group showed that all intrahepatic lesions were hypervascular, with rich tumor staining in the parenchymal phase, appearing as masses or nodules. One case had a hepatic artery-portal vein fistula; no hepatic artery-hepatic vein fistula was observed. Twelve cystic lesions showed faint ring-like tumor staining in the center. Follow-up CT/MRI 5–7 days post-intervention showed significant tumor necrosis, partially presenting as honeycomb-like necrosis. Two months after treatment, in the observation group, 10 patients achieved CR, 22 achieved PR, and 2 had SD, with no cases of liver metastasis progression. The ORR was 94.12%, and the DCR was 100%. In the control group, 2 patients achieved PR, 15 had SD, and 26 had PD. The ORR was 4.65%, and the DCR was 39.53%. Both ORR and DCR were significantly higher in the observation group than in the control group (P < 0.001) (**Table 2**).

**Table 2:** Comparison of short-term clinical efficacy between the two groups [n (%)].

Group	CR	PR	SD	PD	ORR	DCR
Observation (n=34)	10 (29.41%)	22 (64.71%)	2 (5.88%)	0 (0%)	32 (94.12%)	34 (100.00%)
Control (n=43)	0 (0%)	2 (4.65%)	15 (34.88%)	26 (60.47%)	2 (4.65%)	17 (39.53%)

CR, complete response; PR, partial response; SD, stable disease; PD, progressive disease; ORR, objective response rate; DCR, disease control rate.

### 3.2 Survival analysis

**Figure 1:**
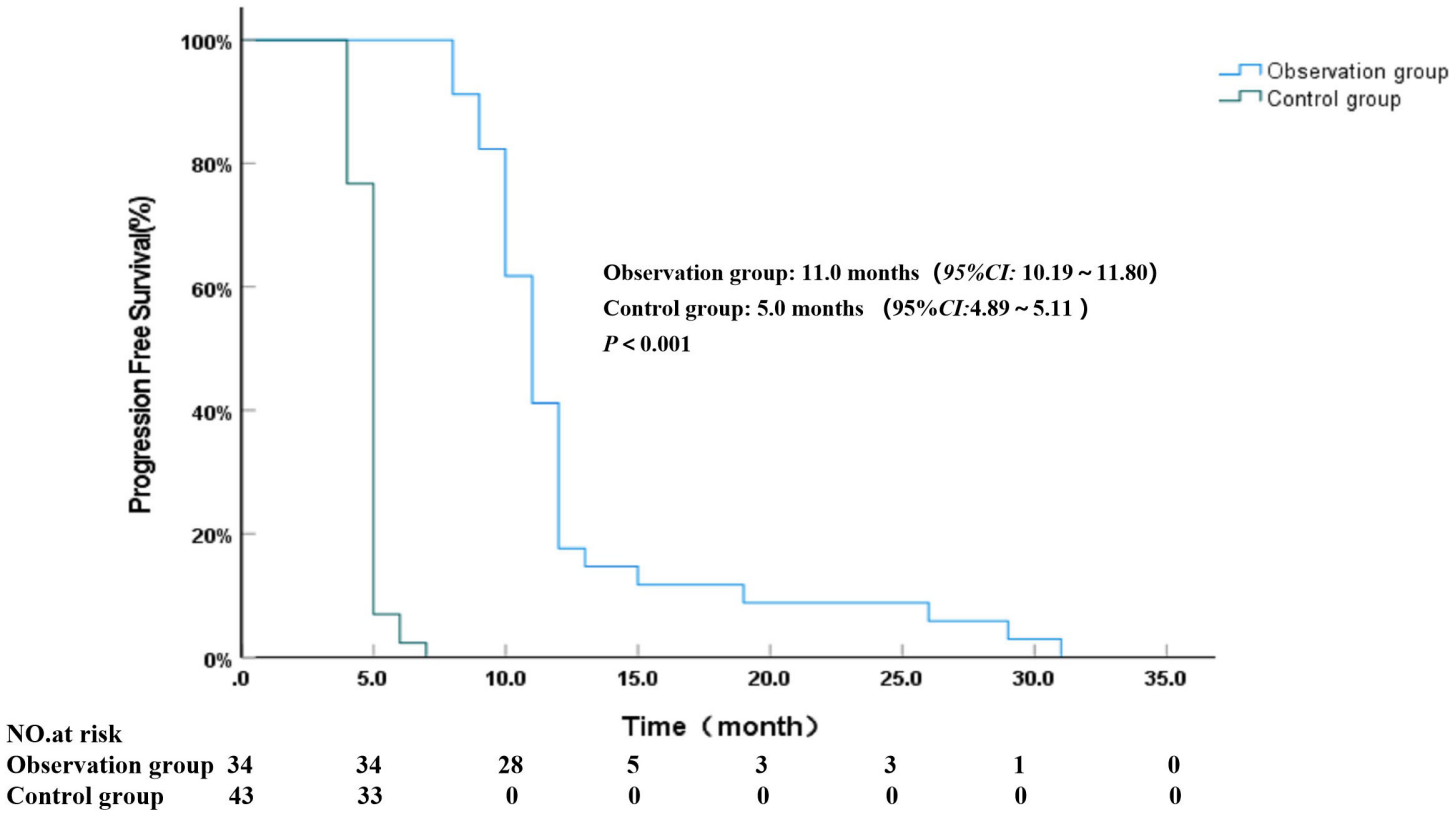
PFS in the observation group and control group.

**Figure 2:**
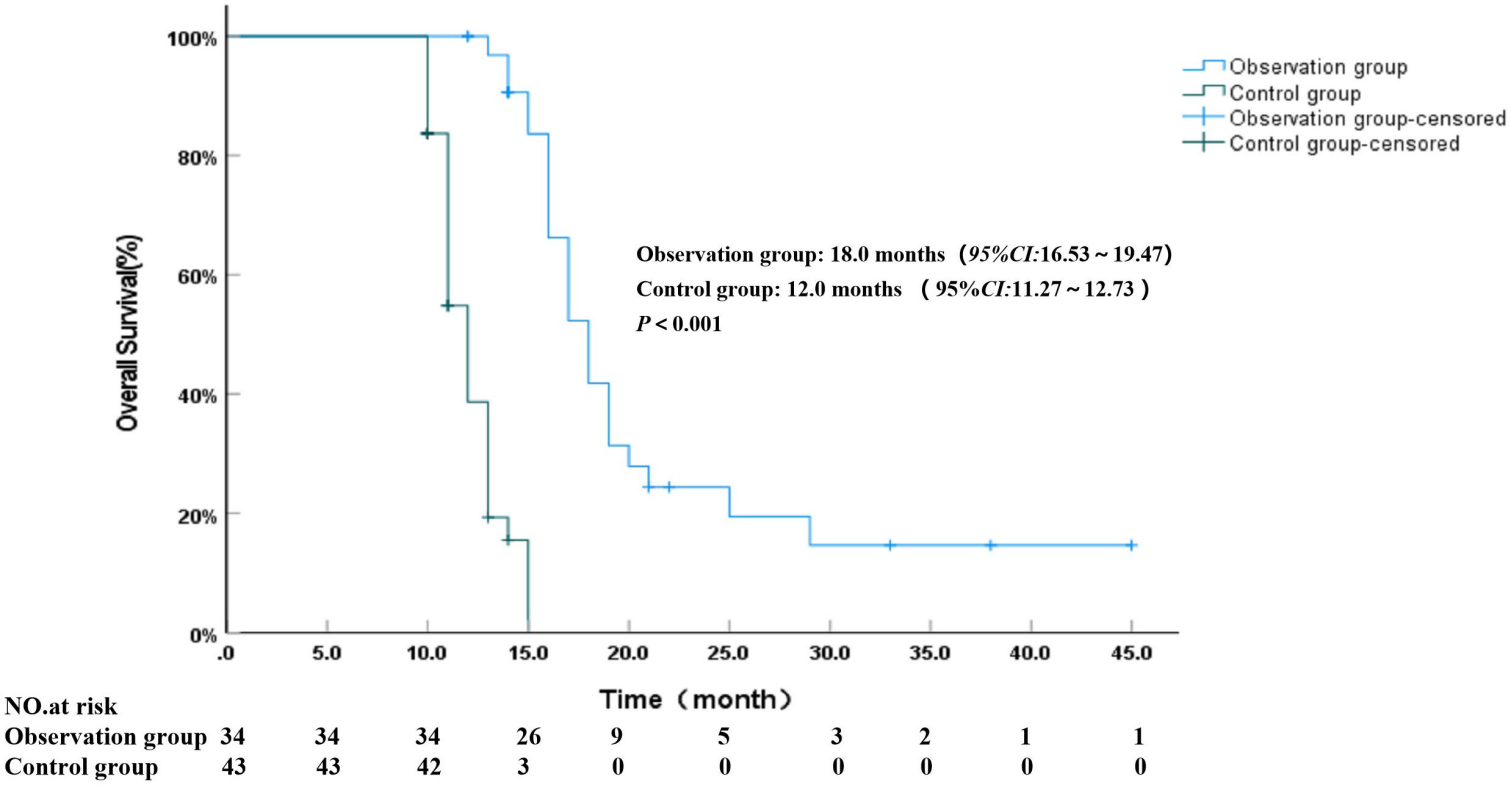
OS in the observation group and control group.

As of the follow-up cutoff date (October 31, 2025), the follow-up period ranged from 10 to 45 months, with a median follow-up of 13 months (mean 14.83 ± 6.17 months). No patients were lost to follow-up. At the end of follow-up, the median PFS was 11.0 months (95% CI: 10.19~11.80) in the observation group and 5.0 months (95% CI: 4.89~5.11) in the control group, a statistically significant difference (P < 0.001). The median OS was 18.0 months (95% CI: 16.53~19.47) in the observation group and 12.0 months (95% CI: 11.27~12.73) in the control group, also a statistically significant difference (P < 0.001) (**Figures 1**, **2**).

### 3.3 Analysis of factors influencing PFS and OS

Univariate analysis showed that Child-Pugh grade, tumor number, number of prior treatment lines, and treatment modality were factors influencing PFS in patients with GIST liver metastases after targeted therapy failure (P < 0.05). Variables with statistically significant differences in univariate analysis were included in the multivariate analysis. Multivariate Cox regression revealed that Child-Pugh grade and treatment modality were independent factors influencing PFS (P < 0.05). Univariate analysis showed that Child-Pugh grade, ECOG score, number of prior treatment lines, and treatment modality were factors influencing OS (P < 0.05). Multivariate analysis indicated that the number of prior treatment lines and treatment modality were independent factors influencing OS (P < 0.05) (**Tables 3**, **4**).

**Table 3:** Univariable and multivariable Cox regression analyses for progression-free survival.

Variable	Category	Univariable analysis		Multivariable analysis	
		HR (95% CI)	P valu	HR (95% CI)	P value
Age (Year)	Per year increase	1.01 (0.99-1.03)	0.307	–	–
Gender	Male (Ref: Female)	1.12 (0.76-1.65)	0.567	–	–
Tumor location	Stomach (Ref)	1.00	–	–	–
	Small intestine	1.25 (0.82-1.91)	0.298	–	–
	Other	1.18 (0.70-1.99)	0.531	–	–
Tumor number	≤3 (Ref)	1.00	–	–	–
	>3	1.60 (1.05-2.44)	0.029	1.35 (0.87-2.09)	0.182
Tumor size (cm)	≤5 (Ref)	1.00	–	–	–
	>5	1.30 (0.88-1.92)	0.184	–	–
Extrahepatic metastasis	No (Ref)	1.00	–	–	–
	Yes	1.28 (0.91-1.79)	0.156	–	–
Child grade	A (Ref)	1.00	–	–	–
	B	1.85 (1.20-2.85)	0.006	1.72 (1.10-2.68)	0.017
ECOG score	0 (Ref)	1.00	–	–	–
	1~2	1.35 (0.90-2.02)	0.144	–	–
Previous treatment lines	Second-line (Ref)	1.00	–	–	–
	Third-line	1.40 (0.90-2.18)	0.132	1.32 (0.84-2.07)	0.226
	Fourth-line	1.55 (1.02-2.36)	0.040	1.42 (0.92-2.19)	0.113
Combination therapy	No (Ref)	1.00	–	–	–
	Yes(TACE combined)	0.45 (0.32-0.63)	<0.001	0.48 (0.34-0.68)	<0.001

HR, hazard ratio; CI, confidence interval; Ref, reference category; TACE, transarterial chemoembolization.

**Table 4:** Univariable and multivariable Cox regression analyses for overall survival.

Variable	Category	Univariable analysis		Multivariable analysis	
		HR (95% CI)	P value	HR (95% CI)	P value
Age (Year)	Per year increase	1.01 (0.99-1.03)	0.307	–	–
Gender	Male (Ref: Female)	1.12 (0.76-1.65)	0.567	–	–
Tumor location	Stomach (Ref)	1.00	–	–	–
	Small intestine	1.25 (0.82-1.91)	0.298	–	–
	Other	1.18 (0.70-1.99)	0.531	–	–
Tumor number	≤3 (Ref)	1.00	–	–	–
	>3	1.30 (0.88-1.92)	0.184	–	–
Tumor size (cm)	≤5cm (Ref)	1.00	–	–	–
	>5cm	1.30 (0.88-1.92)	0.184	–	–
Extrahepatic metastasis	No (Ref)	1.00	–	–	–
	Yes	1.28 (0.91-1.79)	0.156	–	–
Child grade	A (Ref)	1.00	–	–	–
	B	1.80 (1.15-2.82)	0.010	1.44 (0.91-2.29)	0.116
ECOG score	0 (Ref)	1.00	–	–	–
	1~2	1.75 (1.12-2.74)	0.014	1.42 (0.90-2.25)	0.130
Previous treatment lines	Second-line (Ref)	1.00	–	–	–
	Third-line	1.45 (0.93-2.26)	0.102	1.38 (0.88-2.16)	0.158
	Fourth-line	1.95 (1.20-3.16)	0.007	1.80 (1.10-2.95)	0.019
Combination therapy	No (Ref)	1.00	–	–	–
	Yes(TACE combined)	0.50 (0.35-0.71)	<0.001	0.53 (0.37-0.76)	<0.001

HR, hazard ratio; CI, confidence interval; Ref, reference category; TACE, transarterial chemoembolization.

### 3.4 Adverse events

The spectrum of adverse events differed between the two groups, as detailed in **Table 5**. The observation group exhibited a significantly higher incidence of fever, abdominal pain, and nausea/vomiting, which were consistent with the expected symptoms of post-embolization syndrome following DEB-TACE. In contrast, the incidence of typical targeted therapy-related events, such as fatigue, edema, diarrhea, and rash, was comparable between the two groups. The rate of elevated transaminases was higher in the observation group, which was likely attributable to the transient hepatic injury associated with the DEB-TACE procedure. The incidence of any adverse event and Grade ≥3 severe adverse events was not significantly different between the two groups.

**Table 5:** Comparison of adverse events between the two treatment groups [n(%)].

Adverse event	Observation group (n=34)	Control group (n=43)	P value
Any Adverse Event	32 (94.12%)	38 (88.37%)	0.456
Grade ≥3 adverse events	8 (23.53%)	9 (20.93%)	0.789
Specific events			
Fever	25 (73.53%)	5 (11.63%)	<0.001
Abdominal/Hepatic pain	22 (64.71%)	3 (6.98%)	<0.001
Nausea/Vomiting	18 (52.94%)	7 (16.28%)	0.001
Fatigue	20 (58.82%)	22 (51.16%)	0.499
Edema	10 (29.41%)	15 (34.88%)	0.637
Diarrhea	8 (23.53%)	9 (20.93%)	0.789
Rash	6 (17.65%)	8 (18.60%)	>0.999
Transaminase elevation	12 (35.29%)	5 (11.63%)	0.023
Leukopenia	5 (14.71%)	6 (13.95%)	>0.999

Grade ≥3 adverse events were graded according to the National Cancer Institute Common Terminology Criteria for Adverse Events (CTCAE) version 5.0.

## 4 Discussion

Tyrosine kinase inhibitors (TKIs), particularly imatinib, have marked a milestone in the treatment of metastatic gastrointestinal stromal tumors (GIST), significantly prolonging overall survival in these patients (9–12). However, acquired resistance has emerged as a major therapeutic challenge. Disease progression characterized by liver metastases, in particular, leads to a rapid increase in tumor burden and subsequent clinical deterioration, posing substantial difficulties for clinicians (13). Recent studies suggest that combining local therapies with TKIs can rapidly reduce tumor burden, effectively delay the onset of acquired resistance and disease progression, and thereby extend the therapeutic window for TKI treatment (14–16). Based on this concept, local modalities such as surgical resection, radiofrequency ablation, and transarterial chemoembolization (TACE) have been widely integrated into the multidisciplinary management of GIST liver metastases, becoming crucial strategies for improving patient outcomes. Compared with TKI monotherapy, TKI combined with surgical resection has been shown to further improve overall survival (OS) (17). In selected patient populations, radiofrequency ablation can achieve survival benefits comparable to surgical resection (18). TACE has also been demonstrated to be a safe and effective approach after TKI failure (19). A study by Cao et al. reported that TACE using Embosphere microspheres resulted in higher tumor response rates and OS compared to conventional lipiodol-based TACE in patients with imatinib-resistant GIST liver metastases (20), suggesting the potential superiority of microsphere embolics in TACE for GIST liver metastases. Our previous research has confirmed that DEB-TACE yields higher tumor response rates in both primary and metastatic liver cancer (21–23). Nevertheless, to our knowledge, no studies have specifically investigated DEB-TACE for the treatment of GIST liver metastases to date.

The findings of this study confirm that DEB-TACE combined with targeted therapy or other comprehensive treatments, compared to medical therapy alone (TKI/BSC), significantly improves short-term efficacy and prolongs both progression-free survival (PFS) and overall survival (OS), offering a new therapeutic strategy for managing TKI resistance. Furthermore, through multivariate prognostic analysis, this study further delineates the characteristics of patients who are most likely to benefit from this treatment strategy. The multivariate analysis identified the treatment modality (i.e., whether DEB-TACE was combined) as the strongest independent predictor for both PFS and OS, demonstrating a highly significant protective effect. Child-Pugh grade was also confirmed as an independent factor influencing PFS, while the number of prior lines of therapy was an independent factor for OS. These results suggest that patients with preserved liver function may achieve greater survival benefit from DEB-TACE when it is incorporated at an earlier line of therapy. It is worth noting that although”tumor number”was a significant predictor in the univariate analysis for PFS, it lost statistical significance after multivariate adjustment. This is likely attributable to collinearity with other covariates, particularly Child-Pugh grade and treatment modality, which emerged as stronger independent predictors. This finding suggests that the prognostic impact of tumor burden may be at least partially mediated through its association with liver function and treatment selection. In contrast, “prior lines” remained an independent predictor for OS in the multivariate analysis, indicating that cumulative drug resistance and disease aggressiveness, as reflected by the number of prior lines of therapy, exert a more direct and independent influence on long-term survival.

The mechanism underlying the observed survival advantage remains unclear but may be related to the precise targeting of resistant clone cells by DEB-TACE. When GIST develops resistance to TKIs, liver metastases often develop highly hypervascular “tumor-within-a-tumor” areas, which are considered colonies of resistant cells driving disease progression (24). DEB-TACE capitalizes on the hypervascular nature of liver tumors by using tumor-feeding vessels to deliver and maintain high concentrations of chemotherapeutic agents precisely at the lesion core, while simultaneously embolizing these feeding vessels. This dual attack of “local high-dose chemotherapy” and “devascularization” can rapidly reduce hepatic tumor burden (25, 26). From the perspective of tumor evolutionary dynamics, this approach may reduce tumor heterogeneity, delay the emergence of resistant subclones, and create a favorable tumor microenvironment for subsequent reintroduction of TKI therapy (27–30). We postulate that this may constitute the core biological rationale for the efficacy of this combination treatment strategy.

In our study, adverse events in the observation group were primarily those associated with DEB-TACE, such as post-embolization syndrome (e.g., fever, hepatic pain, nausea, and vomiting). Although the incidence was higher than in the control group, the vast majority were Grade 1–2 and resolved rapidly with symptomatic management. Furthermore, no serious complications such as liver abscess, cholecystitis, or non-target embolization occurred in the observation group. Additionally, we found no significant differences between the two groups in the incidence of targeted therapy-related toxicities (e.g., fatigue, diarrhea, rash) or Grade ≥3 severe adverse events. This indicates that the addition of DEB-TACE did not confer significant additional risk. Moreover, after achieving effective control of intrahepatic lesions, this combination strategy may suggest a potential”treatment holiday”effect,allowing for the temporary suspension of subsequent systemic therapy while maintaining disease stability. This hypothesis-generating observation warrants further validation in prospective studies with pre-specified criteria.

Despite the favorable clinical outcomes, this study has several limitations. First, the retrospective design introduces the potential for selection bias. Second, the inherent heterogeneity in the “reintroduced therapy” regimens, while reflective of real-world clinical practice, may affect the consistency of result interpretation. These limitations highlight directions for future research. Firstly, prospective, multicenter, randomized controlled trials are urgently needed to provide higher-level evidence and clarify the role of DEB-TACE in this patient population. Secondly, future efforts should focus on precision medicine to identify the patient subgroup most likely to benefit from DEB-TACE. This could involve analyzing primary and secondary genetic mutation profiles, utilizing dynamic contrast-enhanced imaging or radiomic features to non-invasively assess tumor vascularity and heterogeneity, and exploring the predictive and monitoring value of liquid biopsy markers such as circulating tumor DNA. The ultimate goal is to develop individualized treatment decision models to maximize the clinical benefit of this combination strategy for suitable patients.

In conclusion, the results of this study demonstrate that DEB-TACE can significantly improve the prognosis of patients with TKI-resistant GIST liver metastases, providing a precise and effective treatment option after TKI failure and offering a new therapeutic approach to enhance survival outcomes and quality of life. Nonetheless, prospective, multicenter, randomized controlled studies are warranted to further validate its efficacy and safety.

## Data Availability

The original contributions presented in the study are included in the article/supplementary material. Further inquiries can be directed to the corresponding authors.
